# Identification of p90 Ribosomal S6 Kinase 2 as a Novel Host Protein in HBx Augmenting HBV Replication by iTRAQ‐Based Quantitative Comparative Proteomics

**DOI:** 10.1002/prca.201700090

**Published:** 2018-02-06

**Authors:** Li‐Bo Yan, You‐Jia Yu, Qing‐Bo Zhang, Xiao‐Qiong Tang, Lang Bai, FeiJun Huang, Hong Tang

**Affiliations:** ^1^ Center of Infectious Diseases West China Hospital Sichuan University Chengdu P. R. China; ^2^ Department of Forensic Pathology Medical School of Basic and Forensic Sciences Sichuan University Chengdu China

**Keywords:** HBx protein, hepatitis B virus, p90 ribosomal S6 kinase 2, proteomics, replication

## Abstract

**Purpose:**

The aim of this study was to screen for novel host proteins that play a role in HBx augmenting Hepatitis B virus (HBV) replication.

**Experimental design:**

Three HepG2 cell lines stably harboring different functional domains of HBx (HBx, HBx‐Cm6, and HBx‐Cm16) were cultured. ITRAQ technology integrated with LC‐MS/MS analysis was applied to identify the proteome differences among these three cell lines.

**Results:**

In brief, a total of 70 different proteins were identified among HepG2‐HBx, HepG2‐HBx‐Cm6, and HepG2‐HBx‐Cm16 by double repetition. Several differentially expressed proteins, including p90 ribosomal S6 kinase 2 (RSK2), were further validated. RSK2 was expressed at higher levels in HepG2‐HBx and HepG2‐HBx‐Cm6 compared with HepG2‐HBx‐Cm16. Furthermore, levels of HBV replication intermediates were decreased after silencing RSK2 in HepG2.2.15. An HBx‐minus HBV mutant genome led to decreased levels of HBV replication intermediates and these decreases were restored to levels similar to wild‐type HBV by transient ectopic expression of HBx. After silencing RSK2 expression, the levels of HBV replication intermediates synthesized from the HBx‐minus HBV mutant genome were not restored to levels that were observed with wild‐type HBV by transient HBx expression.

**Conclusion and clinical Relevance:**

Based on iTRAQ quantitative comparative proteomics, RSK2 was identified as a novel host protein that plays a role in HBx augmenting HBV replication.

## 1 Introduction

Hepatitis B virus (HBV) is the prototypic member of the *Hepadnaviridae* family, which contains a group of closely related hepatotropic small DNA viruses with pronounced host specificity.^1^ The HBV genome is a 3.2 kb, circular, partially double‐stranded DNA with four open reading frames (ORFs) named pre‐C/C, pre‐S/S, P, and X. The pre‐C/C ORF encodes the hepatitis B e antigen (HBeAg) and hepatitis B core antigen (HBcAg); the pre‐S/S ORF encodes the hepatitis B surface antigen (HBsAg); the P gene encodes the viral polymerase; the X gene encodes a nonstructural protein known as hepatitis B virus X‐protein (HBx).^2^ HBx is a 154‐amino acid protein with an N‐terminal negative regulatory domain and a C‐terminal transactivation or coactivation domain. HBx is a multifunctional regulator that modulates gene transcription, signaling pathways, genotoxic stress responses, protein degradation, cell cycle, cell proliferation, and apoptosis and genetic stability by directly or indirectly interacting with host factors.^3^

The role of HBx in the viral life cycle has been investigated by several studies. It has been demonstrated that X‐deficient hepadnaviral genomes inoculated into woodchuck livers are unable to initiate productive infections.^4^ Moreover, the role of HBx in activating HBV transcription and replication has been recently demonstrated using cellular systems and mouse models. HBx could restore HBV transcription and replication with the X‐deficient replicon to wild‐type levels in vivo and in vitro.^1^, ^5^, ^6^, ^7^ To further investigate the precise regions of HBx involved in the stimulation of HBV transcription and replication, a study performed by Tang et al. constructed a series of clustered alanine substitution mutants (Cm1–Cm21) of HBx using an alanine scanning mutagenesis strategy. For each mutant, seven consecutive amino acids were replaced by the sequence AAASAAA. The HBx mutants Cm1–Cm7 and Cm10–Cm12 (2–50 aa, 67–87 aa) retained the ability to complement the augmentation effect of HBx on HBV replication. HBx mutants Cm8–Cm9 and Cm13–Cm21 (52–65 aa, 88–154 aa) were unable to restore the augmentation function of HBx.^7^, ^8^ It was also found that the transactivation and coactivation activities of HBx coincide well with its augmentation function in HBV transcription and replication.^7^ Therefore, the regions spanning aa 52–65 and aa 88–154 of HBx are important for the transactivation or coactivation activities of HBx and its stimulatory function in HBV transcription and replication.

**Clinical relevance**

HBx plays an important role in augmenting HBV replication by the transcriptional transactivation function in vivo and in vitro. However, the underlying molecular mechanism by which HBx enhances HBV replication is not fully understood. The transcriptional transactivation function of HBx is dependent on pleiotropic protein–protein interactions. The “key” host proteins involved in HBx enhancement of HBV transcription and replication have not been identified. Clarification of the roles of the host proteins in HBx argumentation of HBV transcription and replication would help to elucidate the HBV pathogenicity mechanism. In this study, we utilized iTRAQ proteomic methodology coupled with LC‐ESI‐MS/MS to identify and quantitate differentially expressed proteins among HepG2 cell lines stably harboring different functional domains of HBx. RSK2 was identified as a novel host protein that plays a role in HBx augmenting HBV replication based on iTRAQ quantitative comparative proteomics. This result may help to better understand the HBV pathogenicity mechanism and may provide new therapy targets for HBV replication.

Although the first activity identified for HBx was the ability to activate transcription of viral and cellular genes, HBx is unable to directly bind to any HBx‐responsive elements in viral and host genes. The trans‐acting transcriptional activity of HBx in enhancing HBV replication may depend on protein–protein interactions. The regulation of transactivation activity by HBx relies on the interactions with several components of the basal transcriptional machinery, cellular sequence‐specific transcription factors, or activation of signal transduction pathways.^9^ However, the underlying molecular mechanism of HBx activating HBV replication are not fully understood. The “key” host proteins involved in HBx activating HBV transcription and replication are not fully understood.

Isobaric tags for relative and absolute quantitation (iTRAQ) coupled with liquid chromatography coupled with tandem mass spectrometry (LC‐MS/MS) is a powerful proteomics technique for quantifying protein changes. ITRAQ technology has many advantages compared with 2D gel electrophoresis, including high‐throughput capabilities and identification of low‐abundance proteins. The advantages of iTRAQ have made it applicable for investigating the underlying molecular mechanisms in many types of scientific research.

To elucidate the molecular mechanisms of HBx regulation of HBV replication, we utilized iTRAQ proteomic methodology coupled with LC‐MS/MS to identify and quantitate proteins that are differentially expressed in HepG2 cells lines stably harboring different functional domains of HBx. In addition, our approach led to the identification of p90 ribosomal S6 kinase 2 (RSK2), which was found to be highly expressed in HepG2 cell lines stably harboring functional domain of HBx. In addition, the role of RSK2 in HBx enhancement of HBV transcription and replication was investigated.

## 2 Experimental Section

### 2.1 Plasmid Constructions

The plasmid payw1.2 (1.2wt, subtype ayw) has been described previously,^7^ and contains 1.2 copies of the wild‐type HBV genome. The HBx‐minus mutant vector payw*7(1.2x(‐)) contains 1.2 copies of HBx‐minus HBV genome.^7^

The mammalian expression plasmids pNKF‐HBx expresses full‐length HBx (aa 1–154). Alanine scanning mutagenesis was employed to construct a series of clustered alanine substitution mutants (designated Cm) as previously described.^7^ The pNKF‐Xcm6 plasmid that expresses HBx with a 7‐aa residue mutant retained the ability to complement the augmentation effect of HBx (aa 37–43 was changed to AAASAAA). The pNKF‐Xcm16 plasmid that expresses HBx with a 7‐aa residue mutant was unable to complement the augmentation effect of HBx (aa 101–108 changed to AAASAAA).

The mammalian expression plasmids pcDNA3.1‐HBx, pcDNA3.1‐Xcm6, and pcDNA 3.1‐Xcm16 were derived from pNKF‐HBx, pNKF‐Xcm6, and pNKF‐Xcm16, respectively. The forward primer contained an EcoRI site (5’‐TACGAATTCATGGCTGCTAGGG TGTGC‐3’), and the reverse primer contained an Xba I site (5’‐GCGTCTAGATTAGGCAGAGGTGAAAAAGTTGC‐3’).

### 2.2 Cell Culture

The human hepatocellular carcinoma HepG2 cells were cultured in DMEM with 10% fetal bovine serum, 1 mM glutamate, and100 units mL^−1^ penicillin and were maintained at 37 °C in a 5% CO_2_–air mixture incubator. The stable HBV replication cell line HepG2.2.15 was cultured in DMEM with 100 μg mL^−1^ G418. HepG2 cells that constitutively express X‐wt, X‐cm6, and X‐cm16 were prepared by transfection with pcDNA3.1‐HBx, pDNA3.1‐Xcm6 and pDNA3.1‐Xcm16, respectively. Stably transfected cells were selected in the presence of 400 μg mL^−1^ geneticin for 2–3 weeks. HepG2‐HBx‐Cm6 retained the ability to complement the augmentation effect, whereas HepG2‐HBx‐Cm16 did not.

### 2.3 Protein Sample Preparation

The cells were suspended in the lysis buffer (7 M urea, 2 M thiourea, 4% CHAPS, 40 mM tris‐HCl, pH 8.5, 1 mM PMSF, and 2 mM EDTA) and were sonicated on ice. The proteins were reduced with 10 mM DTT (final concentration) at 56 °C for 1 h and then were alkylated with 55 mM IAM (final concentration) in the dark for 1 h. The reduced and alkylated protein mixtures were precipitated by adding a 4 × volume of chilled acetone and incubating at −20 °C overnight. After centrifugation at 30 000 g at 4 °C, the pellet was dissolved in 0.5 M TEAB (Applied Biosystems, Milan, Italy) and sonicated on ice. After centrifugation at 30 000 g at 4 °C, an aliquot of the supernatant was taken for determination of protein concentration. The proteins in the supernatant were kept at −80 °C for further analysis.

### 2.4 iTRAQ Labeling and SCX Fractionation

Total protein (100 μg) each sample was digested with Trypsin Gold (Promega, Madison, WI, USA) at 37 °C for 16 h with the ratio of protein:trypsin = 30:1. After trypsin digestion, peptides were dried by vacuum centrifugation. Peptides were reconstituted in 0.5 M TEAB and processed according to the manufacture's protocol with 8‐plex iTRAQ reagent (Applied Biosystems). Briefly, one unit of iTRAQ reagent was thawed and reconstituted in 24 μL isopropanol. Samples were labeled with the iTRAQ tags as follows: Sample X‐wt (119,121 tag), Sample X‐Cm6 (113,115 tag), and X‐Cm16 (116,117 tag). Peptides were labeled with the isobaric tags, and were incubated at room temperature for 2 h. The labeled peptide mixtures were then pooled and dried by vacuum centrifugation.

SCX chromatography was performed with an LC‐20AB HPLC Pump system (Shimadzu, Kyoto, Japan). The iTRAQ‐labeled peptide mixtures were reconstituted with 4 mL buffer A (25 mM NaH_2_PO_4_ in 25% ACN, pH 2.7) and loaded onto a 4.6 × 250 mm Ultremex SCX column containing 5 μm particles (Phenomenex). The peptides were eluted at a flow rate of 1mL min^−1^ with a gradient of buffer A for 10 min, 5–60% buffer B (25 mM NaH_2_PO_4_, 1 M KCl in 25% ACN, pH 2.7) for 27 min, 60–100% buffer B for 1 min. The system was then maintained at 100% buffer B for 1 min before equilibrating with buffer A for 10 min prior to the next injection. Elution was monitored by measuring the absorbance at 214 nm, and fractions were collected every 1 min. The eluted peptides were pooled into 20 fractions, desalted with a Strata X C18 column (Phenomenex) and vacuum dried.

### 2.5 LC‐ESI‐MS/MS Analysis Based on Triple TOF 5600

Each fraction was resuspended in buffer A (5% ACN, 0.1%FA) and centrifuged at 20 000 g for 10 min; the final concentration of peptide averaged approximately 0.5 μg μl^−1^. And 10 μL supernatant was loaded onto an LC‐20AD nano HPLC (Shimadzu, Kyoto, Japan) by the autosampler onto a 2 cm C18 trap column. Peptides were eluted onto a 10 cm analytical C18 column (inner diameter 75 μm) packed in‐house. The samples were loaded at 8 μL min^−1^ for 4 min, then the 35 min gradient was run at 300 nL min^−1^ starting from 2 to 35% B (95%ACN, 0.1%FA), followed by 5 min linear gradient to 60%, then, followed by 2 min linear gradient to 80%, and maintenance at 80% B for 4 min, and finally return to 5% for 1 min.

Data acquisition was performed with a TripleTOF 5600 System (AB SCIEX, Concord, ON) fitted with a Nanospray III source (AB SCIEX, Concord, ON) and a pulled quartz tip as the emitter (New Objectives, Woburn, MA). Data were acquired using an ion spray voltage of 2.5 kV, curtain gas of 30 psi, nebulizer gas of 15 psi, and an interface heater temperature of 150 °C. The MS was operated with an RP greater than or equal to 30 000 FWHM for TOF MS scans. Information‐dependent acquisition survey scans were acquired at 250 ms and as many as 30 product ion scans were collected if exceeding a threshold of 120 counts per second with a 2+ to 5+ charge state. Total cycle time was fixed at 3.3 s. The Q2 transmission window was 100 Da for 100%. Four‐time bins were summed for each scan at a pulser frequency of 11 kHz by monitoring of the 40 GHz multichannel TDC detector with four‐anode channel detection. A sweeping collision energy setting of 35 ± 5 eV coupled with iTRAQ adjust rolling collision energy was applied to all precursor ions for collision‐induced dissociation. Dynamic exclusion was set for 1/2 of peak width (15 s), and then the precursor was refreshed off the exclusion list.

ITRAQ‐based quantitative comparative proteomics were completed by the Beijing Genomics Institute. Protein identification was performed using the Mascot search engine (Matrix Science, London, UK; version 2.3.02). To reduce the probability of false peptide identification, only peptides with significance scores (≥ 20) at the 99% confidence interval by a Mascot probability analysis greater than “identify” were counted as identified. Each confidence protein identification involves at least one unique peptide.

### 2.6 Bioinformatic Analysis

For protein quantitation, it was required that a protein contains at least two unique peptides. The quantitative protein ratios were weighted and normalized by the median ratio in Mascot. We only used ratios with *p*‐values < 0.05, only fold changes of **>** 1.5, and double repetition was considered significant.

Functional annotations of proteins were conducted using the Blast2 GO program against the nonredundant protein database (NR; NCBI). The KEGG database (http://www.genome.jp/kegg/) and the COG database (http://www.ncbi.nlm.nih.gov/COG/) were used to classify and group identified proteins.

### 2.7 Real‐Time Quantitative RT‐PCR Analysis

Total RNA was extracted using Trizol reagent (Invitrogen, IL, USA). One microgram of RNA was reversed‐transcribed into cDNA with a reverse transcription kit (Takara, Japan). Quantitative RT‐PCR was performed on the LightCycler 96 System (Roche, Germany) and using the FastStart Essential DNA Green Master (Roche, Germany) according to the manufacturer's instructions. Primers are listed in Table 1.

**Table 1:** Primer sequences used in the quantitative RT‐PCR analysis

Gene	Forward primer	Reverse primer
ADH4	5’‐TCCAGAGGAGCTA ATAATCGG‐3’	5’‐AAAGGCAGGGTATGGGTCA‐3’
SOD1	5’‐GGTCCTCACTTTAATCCTCTATC‐3’	5’‐TTCTTCATTTCCACCTTTGC‐3’
CSTB	5’‐TACCAAGACCCA GCCCAACT‐3’	5’‐GCCAAGGCACAGCGTAGAT‐3’
ACSL4	5’‐ GGCATTCCTCCAAGTAGACC‐3’	5’‐CATGA GCCAAAGGCAAGT‐3’
PLIN2	5’‐ GACTGCCTATTC TGAATCAG‐3’	5’‐CACTGCCCCTTTGGTCTTGT‐3’;
APOA1	5’‐GGCATTTCTGGCAGCAAGAT‐3’	5’‐GGAGC TTAGGTTTAGCTGT‐3’
RSK2	5’‐CGTGGCAGAAGATGGCTGTG‐3’	5’‐CTGCCTTTTCATGTCCTTCCT‐3’
PRKAR1A	5’‐AAGGTAGGAGGCGA CGAG‐3’	5’‐GCGATAAAGGAGACCGAA A‐3’
RRP1B	5’‐CTCCAGTTTGACTATAAGGCT‐3’;	5’‐GAGAGGCGCTTCCTGTTGAA‐3’
DPP4	5’‐ AAGGCACCTGGGAAGTCA‐3’	5’‐GCTCACAACTGAGGCAT G‐3’
FABP1	5’‐ GGCAAGTACCAACTGCAGAG‐3’	5’‐ CTTGAAGTGCTTCCCATTCT‐3’
HBcAg	5’‐CTGGGTGGGTGTTAATTTGG‐3’	5’‐TAAGCTGGAGGAGTGCGAAT5’–3’

### 2.8 Western Blotting

The cells were lysed at 4 °C in lysis buffer (20 mM tris‐HCl, pH 7.6, 0.5% NP‐40, 250 mM NaCl, 3 mM EGTA, 3 mM EDTA, 1 mM sodium vanadate,2 mM dithiothreitol, 0.5 mM PMSF, 20 mM β‐glycerophosphate, and 1 μg mL^−1^ leupeptin).

The protein samples (20 μg) were separated by 10% SDS‐polyacrylamide gel electrophoresis and transferred to PVDF membrane, and proteins were detected by Western blot.

### 2.9 Enzyme‐Linked Immunosorbent Assay (ELISA)

HBsAg and HBeAg in the cell culture medium and mouse serum were measured by using ELISA kits (Kehua Bioengineering, Shanghai, China). HepG2.2.15 and HepG2 cells were seeded in 6‐well plates at 70–80% confluence. After the cells were cultured for 24 h, they were transfected as indicated in the Figure4 legend. The culture media was collected, and the levels of HBsAg and HBeAg were measured according to the manufacturer's instructions.

### 2.10 Southern Blotting

The isolation and determination of HBV DNA replication intermediates were performed as previously described. These HBV DNA replication intermediates (HBV DNA RI) were resuspended in 30 μL of tris‐ethylene diamine tetraacetic acid buffer. The samples were separated by 1% agarose and transferred to Hybond‐N+ membrane (Amersham Biosciences, Bucks, UK). The membrane were probed with digoxigenin‐labeled full‐length HBV DNA sequence and then analyzed using the DIG Luminescent Detection Kit for Nucleic Acids (Roche, Germany).

## 3 Results

### 3.1 ITRAQ Analysis of Differentially Expressed Proteins

**Figure 1:**
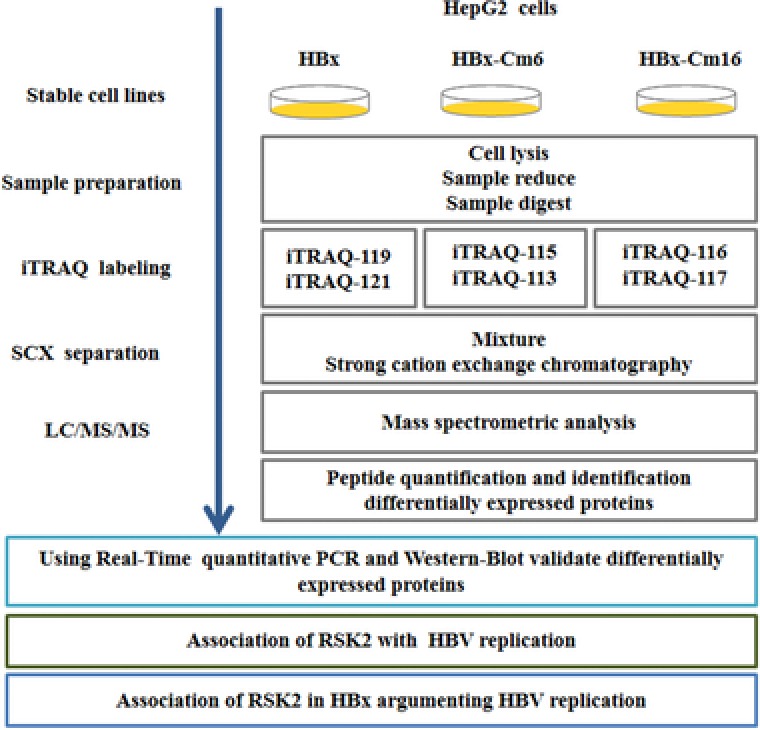
Schematic summary of experimental and data analysis process of the study.

We utilized iTRAQ proteomic methodology coupled with LC‐MS to identify differentially expressed proteins. HepG2 cells lines which harbored different functional domain of HBx were investigated. HepG2‐HBx and HepG2‐HBx‐Cm6 retained the ability to complement the augmentation effect on HBV replication, whereas HepG2‐HBx‐Cm16 did not. To increase the coverage of protein identification and/or the confidence of the data generated, samples were iTRAQ labeled as follows: HepG2‐HBx (119,121 tag), HepG2‐HBx‐Cm6 (113,115 tag), and HepG2‐HBx‐Cm16 (116,117 tag). A schematic flowchart of the iTRAQ method is shown in Figure 1.

**Figure 2:**
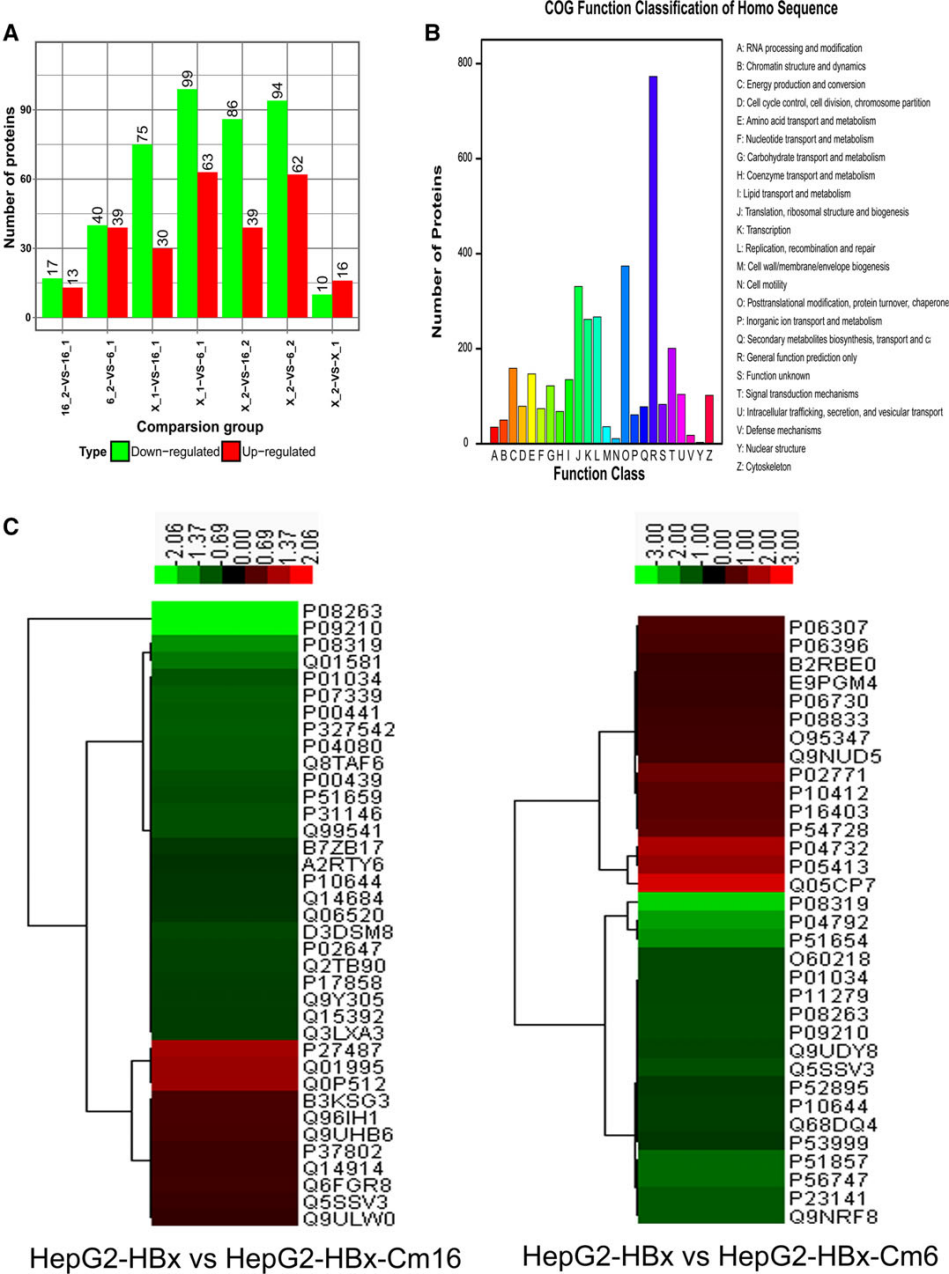
A) The number of differentially expressed proteins. B) Cluster of orthologous groups of proteins. Function classification of Homo Sequence. C) Functional clustering analysis of the identified proteins from HepG2‐HBx, HepG2‐HBx‐Cm6 ,and HepG2‐HBx‐Cm16 by double repetition.

A total of 548 proteins were identified as being differentially expressed (over 1.5‐fold, *p* < 0.05) among three cell lines (Figure 2A). These 548 proteins were classified into 24 functional categories by using cluster of orthologous groups (Figure 2B). To reduce false positives for the selection of differentially expressed proteins, 70 differentially expressed proteins were identified by double repetition among these three groups (119:116,121:117; 119:113,121:115; Tables 2 and 3). Functional clustering analyses of the identified proteins from HepG2‐HBx, HepG2‐HBx‐Cm6, and HepG2‐HBx‐Cm16 cells are presented in Figure 2C.

**Table 2:** iTRAQ analysis of differentially expressed proteins between HepG2‐HBx and HepG2‐HBx‐Cm16

No	Protein description	Gene	Function	Acce. no	Score	Mass	Cov	Fold change
1	Glutathione S‐transferase A1	GST A1	Transferase	P08263	138	33277	19.8	0.131
2	Glutathione S‐transferase A2	GST A2	Transferase	P09210	138	32949	19.8	0.131
3	Alcohol dehydrogenase 4	ADH4	Oxidoreductase	P08319	110	53588	12	0.308
4	Hydroxymethylglutaryl‐CoA synthase	HMGCS1	Lipid metabolic process	Q01581	161	66952	22.5	0.376
5	Cathepsin D	CATD	Lysosome	P07339	1078	52338	41.5	0.461
6	4‐hydroxyphenylpyruvate dioxygenase	HPD	Catabolism	P327542	678	49712	39	0.468
7	Superoxide dismutase	SOD1	Oxidoreductase	P00441	597	19804	22.7	0.473
8	Cystatin‐B	CSTB	Thiol protease inhibitor	P04080	273	14232	24.5	0.479
9	Acyl‐CoA synthetase 4	ACSL4	Lipid metabolism	Q8TAF6	601	93710	23.5	0.48
10	Cystatin‐C	CST3	Thiol protease inhibitor	P01034	254	18451	18.5	0.498
11	Coronin‐1A	CORO1A	Structural component	P31146	114	58675	11.3	0.517
12	Perilipin‐2	PLIN2	Cellular component	Q99541	1119	58312	45.8	0.519
13	Phenylalanine‐4‐hydroxylase	PAH	Oxidoreductase	P00439	127	61773	10.2	0.532
14	Peroxisomal multifunctional enzyme type	HSD17B4	Lipid metabolism	P51659	959	97054	37.1	0.542
15	Formimidoyltransferase cyclodeaminase	FTCD	Histidine metabolism	D3DSM8	234	70749	17.9	0.563
16	Apolipoprotein A‐I	APOA1	Lipid metabolism	P02647	128	37756	22.5	0.586
17	Putative hexokinase	HKDC1	Glycolysis transferase	Q2TB90	103	123259	7.1	0.59
18	6‐phosphofructokinase, liver type	PFKL	Glycolysis transferase	P17858	803	97322	24	0.6
19	Acyl‐coenzyme A thioesterase 9	ACOT9	Acyl‐CoA metabolic process	Q9Y305	198	54973	26.1	0.605
20	Dihydroxyacetone kinase	DAK	Kinase, transferase	Q3LXA3	1331	68074	35	0.61
21	Delta(24)‐sterol reductase	DHCR24	Lipid metabolism	Q15392	151	70538	12	0.612
22	Ribosomal protein S6 kinase alpha‐3	RSK2	Protein kinase	B7ZB17	1282	97341	34.9	0.629
23	Bile salt sulfotransferase	SULT2A1	Lipid metabolism	Q06520	193	41534	31.9	0.641
24	cAMP‐dependent protein kinase type I	PRKAR1A	Signal pathway	P10644	523	50180	29.9	0.65
25	Ribosomal RNA processing 1B	RRP1B	Poly(A) RNA binding	Q14684	329	105587	9.1	0.65
26	Inter‐alpha (globulin) inhibitor H2	ITIH2	Serine protease inhibitor	A2RTY6	381	126903	17.8	0.665
27	Targeting protein for Xklp2	TPX2	Apoptosis	Q9ULW0	211	116648	13.8	1.518
28	Dimethylarginine hydrolase 2	DDAH2	Hydrolase	Q5SSV3	221	21790	22.9	1.592
29	Transgelin‐2	TAGLN2	Epithelial cell differentiation	P37802	186	26503	38.2	1.638
30	Prostaglandin reductase 1	PTGR1	Oxidoreductase	Q14914	166	41620	14.6	1.646
31	Major prion protein	PRNP	Prion, cell cycle	Q6FGR8	82	31156	11.9	1.68
32	LIM domain and actin binding 1	LIMA1	Actin binding protein	Q9UHB6	162	108443	14.3	1.793
33	Fascin1	FSCN1	Actin bundling protein	Q96IH1	1622	64551	47.6	1.822
34	Actin binding LIM protein 1	ABLIM1	Actin binding protein	B3KSG3	272	71787	22.8	1.827
35	Transgelin	TAGLN	Epithelial cell differentiation	Q01995	992	28129	65.2	3.526
36	Solute carrier family 2	SLC2A1	Sugar transport	Q0P512	468	59532	12.2	3.568
37	Dipeptidyl peptidase 4	DPP4	Cell adhesion	P27487	251	101379	12.5	3.854

**Table 3:** iTRAQ analysis of differentially expressed proteins between HepG2‐HBx and HepG2‐HBx‐Cm6

No	Protein description	Gene name	Function	Acce. no	Score	Mass	Cov	Fold change
1	Alcohol dehydrogenase 4	ADH4	Oxidoreductase	P08319	110	53588	12	0.182
2	Heat shock protein beta‐1	HSPB1	Molecular chaperone	P04792	438	25259	39	0.274
3	Glypican‐3	GPC3	Protease inhibitor	P51654	154	77193	11.6	0.313
4	Claudin‐6	CLD6	Host–virus interaction	P56747	129	26280	5.5	0.422
5	3‐oxo‐5‐beta‐steroid 4‐dehydrogenase	AKR1D1	Oxidoreductase	P51857	160	44996	8.6	0.425
6	CTP synthase 2	CTPS2	Synthase	Q9NRF8	231	77880	15	0.48
7	Liver carboxylesterase 1	EST1	Transferase	P23141	1014	74630	32.3	0.488
8	Dimethylargininedimethylaminohydrolase 2	DDAH2	Hydrolase	Q5SSV3	221	25551	19	0.527
9	Glutathione S‐transferase A1	GST A1	Transferase	P08263	138	33277	19.8	0.549
10	Glutathione S‐transferase A2	GST A2	Transferase	P09210	138	32949	19.8	0.549
11	Cystatin‐C	CST3	Thiol protease inhibitor	P01034	254	18451	18.5	0.558
12	Aldo‐keto reductase family	AKR1B10	Cellular component	O60218	155	48851	5.1	0.558
13	Lysosomal‐associated membrane protein 1	LAMP1	Host–virus interaction	P11279	155	51147	4.8	0.558
14	Mucosa‐associated lymphoid tissue protein1	MALT1	Ubl conjugation	Q9UDY8	168	108963	10	0.575
15	cAMP‐dependent protein kinase type I	PRKAR1A	Cellular component	P10644	523	50180	29.9	0.601
16	Putative uncharacterized protein	DKFZp 779L0468	Other	Q68DQ4	523	50146	29.9	0.601
17	Aldo‐keto reductase family 1 member C2	AKR1C2	Lipid metabolism	P52895	1325	45325	39.9	0.617
18	SUB1 homolog	SUB1	Transcription	P53999	534	20471	48.8	0.642
19	Eukaryotic translation Initiation factor 4E	EIF4E	Translation	P06730	173	30481	18.4	1.56
20	Long chain fatty acid CoA	ACSL3	Fatty acid metabolism	B2RBE0	532	99518	21.7	1.576
21	Glucan‐branching enzyme	GBE1	Glycogen biosynthetic process	E9PGM4	158	87015	9.1	1.586
22	Insulin‐like growth factor binding protein 1	IGFBP1	Insulin‐like growth factor binding	P08833	286	31954	13.1	1.648
23	Structural maintenance of chromosomes protein 2	SMC2	Nucleotide binding	O95347	300	180803	15.5	1.681
24	Zinc finger CCHC domain containing 3	ZCCHC3	Poly(A) RNA binding	Q9NUD5	286	52299	18.6	1.705
25	Gelsolin	GSN	Calcium ion binding	P06396	237	94869	22.2	1.798
26	Cholecystokinin	CCK	Hormone	P06307	104	13688	7.8	1.875
27	Histone H1.2	HIST1H1c	Chromatin DNA binding protein	P16403	1508	39604	30	2.042
28	Histone H 1.4	HIST1H1E	Chromatin DNA binding protein	P10412	1488	41017	32.9	2.042
29	RAD23 homolog B	RAD23B	UV excision repair protein	P54728	370	49590	23.2	2.193
30	Alpha‐fetoprotein	AFP	Secreted protein	P02771	350	85062	16.2	2.383
31	Fatty acid binding protein 3	FABP3	Transport	P05413	138	19398	34.8	3.393
32	Metallothionein‐1E	MT1E	Zinc ion binding	P04732	71	9887	32.8	4.027
33	Fatty acid binding protein 1	FABP1	Transport	Q05CP7	768	22745	52.2	5.737

### 3.2 Validation of Differentially Expressed Proteins

**Figure 3:**
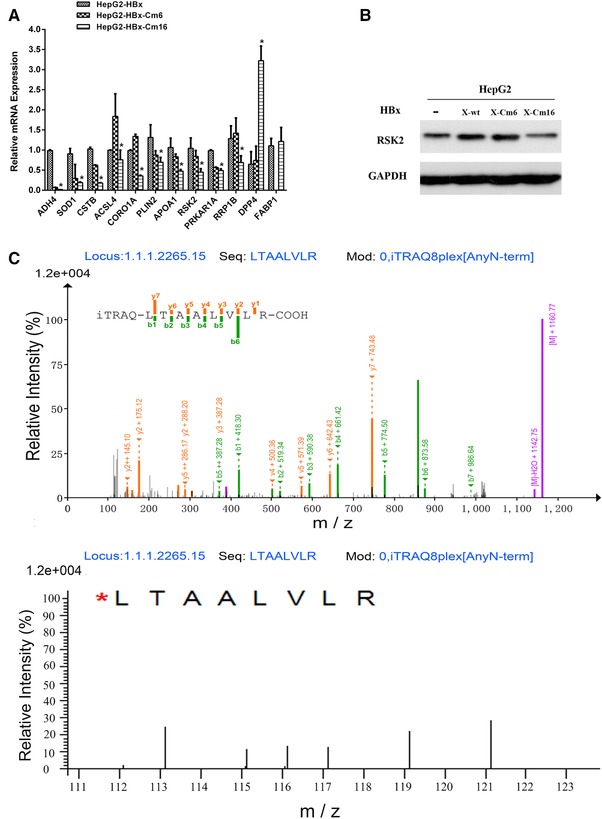
Evaluation of the differentially expressed proteins. A) Real‐time RT‐PCR detected the relative mRNA expression levels of ADH4, SOD1, CSTB, ACSL4, CORO1A, PLIN2, APOA1, RSK2, PRKAR1A, RRP1B, DPP4, and FABP1 in HepG2‐HBx, HepG2‐ HBx‐Cm6, and HepG2‐HBx‐Cm16 cells. * *p *≤ 0.05 differ from HepG2‐HBx and HepG2‐HBx‐Cm16. B) Western blot for RSK2 in cell line HepG2, HepG2‐HBx, HepG2‐HBx‐Cm6, and HepG2‐HBx‐Cm16 cell. C) Representative MS/MS spectrum showing the peptides from RSK2 (peptide sequence: LTAALVLR).

Differential expression levels of the proteins identified by the iTRAQ approach were validated using real‐time quantitative RT‐PCR analysis. Figure 3A shows the relative mRNA expression levels of ADH4, SOD1, CSTB, ACSL4, CORO1A, PLIN2, APOA1, RSK2, PRKAR1A, RRP1B, DPP4, and FABP1.The mRNA levels of ADH4, SOD1, CSTB, ACSL4, CORO1A, PLIN2, APOA1, RSK2, PRKAR1A, and RRP1B were downregulated in HepG2‐HBx‐Cm16 compared with HepG2‐HBx, whereas the mRNA levels of DPP4 and FABP1 were upregulated. These trends were similar with the protein expression levels determined by the iTRAQ approach.

The MS/MS spectrum of p90 RSK2 (peptide sequence: LTAALVLR) is presented in Figure 3C. The ratio of 119:116 and 121:117 indicates the relative abundance of RSK2 protein in HepG2‐HBx compared to that in HepG2‐HBx‐Cm16 cells. Similarly, the ratio of 119:113 and 121:115 indicates the relative abundance of RSK2 protein in HepG2‐HBx compared to that in HepG2‐HBx‐Cm6 cells. When the same protein gave two relative quantitative ratios, the quantitation ratio from the experiment with the best *p*‐values was selected.

Figure 3B shows a representative Western blot analysis of RSK2 expression in four cell lines. RSK2 is expressed at higher levels in HepG2‐HBx and HepG2‐HBx‐Cm6, compared with HepG2 and HepG2‐HBx‐Cm16. HepG2‐HBx‐Cm6 harboring the HBx retained the ability to complement the augmentation effect, whereas HepG2‐HBx‐Cm16 did not. This indicates that the host protein RSK2 might play a role in HBx augmenting HBV replication.

### 3.3 Association of RSK2 With HBV Replication

**Figure 4:**
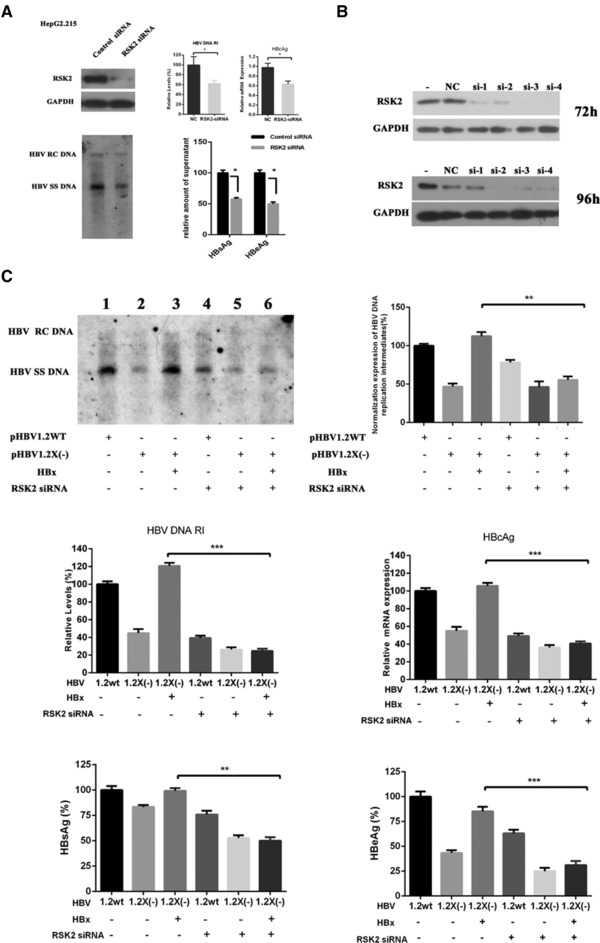
Association of RSK2 in HBV replication and HBx augmenting HBV replication. A) Levels of RSK2, HBV DNA replication intermediates, HBcAg, HBsAg, and HBeAg in HepG2.2.15 and RSK2 siRNA‐transfected HepG2.2.15. B) RSK2 detected by Western blotting after siRNA transfection of HepG2 cells. C) Levels of HBV DNA RI, HBcAg, HBsAg, and HBeAg in HepG2 which were transfected with wild‐type HBV, HBx‐minus HBV, and HBx‐minus HBV plus ectopic expression of HBx plasmid. Data shown are the means ± SD. Statistical significance was examined by one‐way analysis of variance pairwise comparison. *p* < 0.05 was considered statistically significant; *p < 0.05; **p < 0.01; ***p < 0.001. RI: replication intermediates.

RSK2 expression was silenced by siRNA in HepG2 and HepG2.2.15. (Figure 4A,B). As shown by Western blotting, RSK2 siRNA transfection significantly reduced RSK2 protein levels, whereas control siRNA has no effect. The effect of RSK2 siRNA transfection on HBV replication was explored further. The level of HBV replication intermediates in RSK2 siRNA‐transfected HepG2.2.15 cells was twofold lower than that in the controls. The levels of HBV DNA, HBcAg, HBsAg, and HBeAg were decreased 37.4, 34.0, 42.3, and 50.2, respectively, after silencing RSK2 in HepG2.2.15 (Figure 4A). This indicates that silence of RSK2 reduced HBV DNA replication and expression.

### 3.4 Association of RSK2 in HBx Enhancing HBV Replication

As shown by Western blotting in Figure 4A, RSK2 siRNA transfection of HepG2 cells significantly reduced RSK2 protein levels. In HepG2 cells, the levels of HBV DNA replication intermediates synthesized from the HBx‐deficient HBV genome were twofold lower than levels synthesized from the wild‐type HBV genome. Furthermore, the decreased levels of HBV replication intermediates synthesized from the HBx‐minus HBV construct were restored to levels similar to that observed with the wild‐type construct by cotransfection of HBx expression plasmid (two‐ to threefold that of HBx‐deficient HBV). These results indicate that HBx has an augmentation role in HBV transcription and replication. In contrast, the levels of HBV replication intermediates synthesized from HBx‐minus HBV mutant genome were not restored to levels observed with wild‐type HBV by cotransfection of the HBx expression plasmid in RSK2 siRNA‐transfected HepG2 cells. The levels of HBV DNA (*p* < 0.001), HBcAg (*p* < 0.001), HBsAg (*p* < 0.01) and HBeAg (*p* < 0.001) shows the same trend as HBV DNA replication intermediates (Figure 4C).

## 4 Discussion

It has been demonstrated that HBx plays an important role in augmenting HBV replication by transcriptional transactivation function in vivo and in vitro. However, the underlying molecular mechanism by which HBx enhances HBV replication are not fully understood. The transcriptional transactivation function of HBx is dependent on pleiotropic protein–protein interactions. The “key” host proteins involved in HBx enhancement of HBV transcription and replication have not been identified. Clarification of the roles of host proteins in HBx augmentation of HBV transcription and replication would help elucidate the HBV pathogenicity mechanism.

In this study, we utilized iTRAQ proteomic methodology coupled with LC‐ESI‐MS/MS to identify and quantitate differentially expressed proteins among HepG2 cell lines stably harboring different functional domains of HBx. Seventy differentially expressed proteins were identified among HepG2‐HBx, HepG2‐HBx‐Cm6, and HepG2‐HBx‐Cm16 by two duplicate groups. Many of them, including ADH4, SOD1, CSTB, ACSL4, CORO1A, PLIN2, APOA1, RSK2, PRKAR1A, RRP1B, DPP4, and FABP1, were confirmed by qRT‐PCR analysis. RSK2 was expressed at higher levels in HepG2‐HBx and HepG2‐HBx‐Cm6, compared with HepG2‐HBx‐Cm16 and HepG2. These results are consistent with the protein expression level determined by the iTRAQ approach. Tao Zhang et al.^10^ performed an integrated proteomics and bioinformatics analysis of HBx interacting proteins. They also identified RSK2 as a novel interactor. Collectively, these data provide evidence that the iTRAQ reagents labeling method for large scale protein quantification is powerful and reliable for HBV‐related investigations.^11^

The RSK2 siRNA‐transfected HepG2.2.15 cells showed decreased HBV DNA, HBsAg, and HBeAg levels. This indicates that RSK2 is involved in regulating HBV replication. HBx augments HBV replication and expression, because an HBx‐minus HBV mutant genome led to decreased levels of HBV replication intermediates, HBsAg , and HBeAg. These decreases can be restored to levels similar to wild‐type HBV by transient ectopic expression of HBx. After silencing RSK2 expression, the levels of HBV replication intermediates, HBsAg and HBeAg synthesized from the HBx‐minus HBV mutant genome, were not restored to levels observed in wild‐type HBV by transient ectopic expression of HBx. This indicates that RSK2 plays an important role in HBx by augmenting HBV replication. RSK2 was identified as a novel host protein in HBx augmenting HBV replication.

The RSK (90 kDa ribosomal S6 kinase) family comprises a group of highly related serine/threonine kinases that regulate diverse cellular processes, including cell growth, proliferation, survival and motility.^12^ Members of this family, which are downstream effectors of the Ras/ERK signaling pathway, includes four vertebrate isoforms (RSK1, RSK2, RSK3, and RSK4).^13^ RSK2 was identified as an important effector of ERK in global transcriptional regulation. Indeed, activated RSK2 was shown to phosphorylate several transcription factors including AP‐1, CREB, c‐Fos, c‐Jun, and others, some of which contribute to the IEG (immediate‐early gene) response or are IEG products themselves.^14^ Some transcription factors play an important role in the transcription of retrovirus. The previous studies demonstrated that HBx transcriptional activity is also linked to its capacity to stimulate MAPKs and JAK/STAT signaling pathways.^2^ Despite the fact that RSK2 is a member of MAPKs signaling pathways, no study has investigated the relationship between HBx and RSK2. RSK2 has been implicated in other viral infections, such as HIV, HCV, and influenza.^15^, ^16^ Kaposi's sarcoma‐associated herpes virus ORF45 mediates transcriptional activation of the HIV‐1 long terminal repeat via RSK2.^17^, ^18^, ^19^ HIV Tat protein interacts with RSK2 and activated RSK2 kinase activity in cells, and might serve to induce early changes in the chromatin organization of the HIV LTR.^20^ The regulation and function of RSK2 may explain its role in HBx enhancement of HBV replication.

In this study, pathway analysis also revealed that HBx‐related differentially expressed proteins are associated with lipid metabolism. Previous studies indicated that HBx‐induced abnormal lipid metabolism of hepatoma in hepatocarcinogenesis.^21^ This indicates that HBx induces abnormal lipid metabolism to meet the bioenergetic demands of extreme cell growth and proliferation. Another study showed that fatty acids increase HBx stabilization and HBx‐induced inflammatory gene expression.^22^The differentially expressed protein apolipoprotein A‐I (apoA‐I), which has a specific role in lipid and cholesterol metabolism, has been proved to be a novel interactor with HBx and may influence HBV secretion.^10^

In conclusion, RSK2 was identified as a novel host protein in HBx augmenting HBV replication by iTRAQ‐based quantitative comparative proteomics. These results may help to better understand the HBV pathogenicity mechanism.

## Conflict of interest

The authors have declared no conflict of interest.
